# Full ribosomal RNA gene arrays confirm *Marteilia refringens sensu stricto* and *Marteilia pararefringens* as separate species, and assess the validity of current diagnostic regions

**DOI:** 10.1017/S0031182025100796

**Published:** 2025-10

**Authors:** Chantelle Hooper, Delphine Serpin, Isabelle Arzul, Lydie Canier, Mats Bøgwald, Stein Mortensen, Raquel Aranguren, Antonio Figueras, Georgia M Ward, David Bass

**Affiliations:** 1 Centre for Environment, Fisheries and Aquaculture Science (Cefas), Weymouth Laboratory, Weymouth, UK; 2 Centre for Sustainable Aquaculture Futures, University of Exeter, Exeter, UK; 3 Ifremer Adaptation and Santé des Invertébrés Marins-La Tremblade, France; 4 Institute of Marine Research, Bergen, Norway; 5 Institute of Marine Research (IIM), CSIC, Vigo, Spain

**Keywords:** aquatic animal health, bivalves, *Marteilia*, molecular phylogenetics, species delimitation

## Abstract

The species boundary between the paramyxid parasitic protists *Marteilia refringens sensu stricto* and *Marteilia pararefringens* has been disputed, and their classification as separate species has been a topic of debate for the past 2 decades. Originally described as separate species, they were later synonymized based on limited evidence and referred to as 2 types of *M. refringens* (O-type and M-type). In 2018, longer rRNA gene sequences from a small number of samples supported their reclassification as distinct species. However, limited sample sizes and incomplete array coverage left questions regarding the robustness of this separation. We present full transcribed ribosomal RNA (rRNA) gene arrays from a broad set of *Marteilia* samples collected across their known host and geographic ranges. Phylogenetic and species delimitation analysis of these sequences robustly distinguished *M. refringens sensu stricto* from *M. pararefringens*. We identified sites across the entire rRNA array with consistent sequence differences between species and carried out phylogenetic analyses on the most variable regions of the rRNA array (ITS1 and ETS), which also distinguished between the 2 species. We also provide new evidence for distinct host preference profiles for *M. refringens sensu stricto* and *M. pararefringens.* The results support the recognition of *M. refringens sensu stricto* and *M. pararefringens* as separate species and identify robust markers for their detection, allowing a better understanding of their respective ecologies, host preference, pathogenicity and life cycle. The study also lays a foundation for future genomic comparisons to explore evolutionary divergence and diagnostic marker development beyond the rRNA array.

## Graphical abstract

**Figure:**
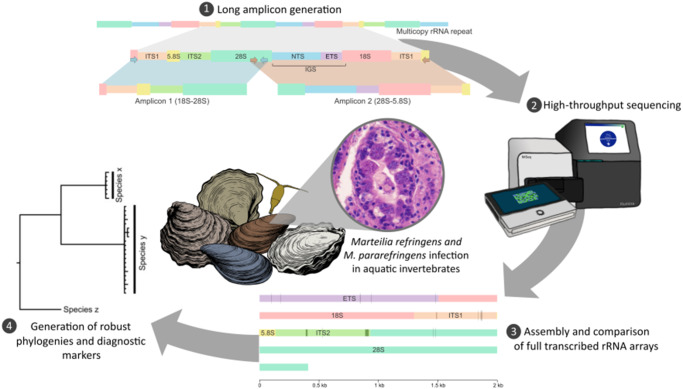

## Introduction

*Marteilia* is a genus of protistan parasites in the order Paramyxida (Rhizaria, Endomyxa, Ascetosporea; Bass et al. (2019)), including species associated with mortalities of aquatic mollusc species around the globe. Characterized by their cell-in-cell morphology, 8 species of *Marteilia* have so far been described and have available molecular sequence data: *M. refringens* (Grizel et al. 1974), *M. pararefringens* (Kerr et al. 2018), *Marteilia sydneyi* (Perkins and Wolf, 1976), *M. cochillia* (Carrasco et al. 2013), *M. (Eomarteilia) granula* (Itoh et al. 2014), *M. octospora* (Ruiz et al. 2016), *M. tapetis* (Kang et al. 2019) and *M. cocosarum* (Skujina et al. 2022). Two other species of *Marteilia* have been described morphologically, but do not have any available sequence data: *M. christenseni* (Comps, 1983) and *M. lengehi* (Comps, 1976). *Marteilia* have a broad geographical range and have been detected in invertebrate hosts in Europe, Oceania, Asia, Africa and the USA (Grizel et al. 1974; Perkins and Wolf, 1976; Moyer et al. 1993; Audemard et al. 2002; Elgharsalli et al. 2013; Adlard and Nolan, 2015).

### Historical taxonomy

Described in the context of mortalities of the flat oyster (*Ostrea edulis*) in 1969, *M. refringens* was the first species of *Marteilia* to be identified (Comps, 1970; Grizel et al. 1974). *Marteilia refringens* is a listed pathogen by both the World Organization for Animal Health (WOAH; previously OIE) (Woah, 2023) and the European Union (under EU regulation 2016/429) as a significant pathogen of bivalve molluscs. Subsequent to the identification of *M. refringens* in flat oysters, a *Marteilia* species was detected in the mussels *Mytilus edulis* (Comps et al. 1975) and *My. galloprovincialis* (Comps et al. 1982). Based on host specificity and ultrastructural characteristics, the *Marteilia* infecting mussels was described as a separate species: *M. maurini* (Comps et al. 1982). Further evidence that the *Marteilia* lineages infecting oysters and mussels were distinct species was provided by Le Roux et al. (2001), who sequenced partial internal transcribed spacer 1 (ITS1) and found consistent nucleotide differences between them. However, Longshaw et al. (2001) challenged the characterization of 2 different species, finding no significant differences in ultrastructure between *M. refringens* and *M. maurini*, albeit based on small sample numbers and limited lifecycle stages. Berthe et al. (2000) also disputed there being 2 species, based on no clear differences in small ribosomal subunit gene (18S) sequence data between *Marteilia* isolated from oysters and mussels. It also became apparent that *M. maurini* could be amplified from *O. edulis*, and *M. refringens* could be amplified from *My. galloprovincialis* (López-Flores et al. 2004; Novoa et al. 2005; Balseiro et al. 2007), so host specificity was disregarded as a species-defining characteristic. It was determined by the European Food Safety Association (European Food Safety, 2008), based on Balseiro et al. (2007), that there was insufficient evidence to maintain separate species status. *Marteilia refringens* and *M. maurini* were therefore synonymized as *M. refringens* in 2007, referred to as ‘O-type’ (previously the ‘oyster-infecting’ *M. refringens*) and ‘M-type’ (previously ‘mussel-infecting’ *M. maurini*), defined by the ITS1 sequence differences identified by Le Roux et al. (2001).

Synonymization of the 2 *Marteilia* species had important consequences – many studies after the synonymization have not distinguished between M and O type (only 28 out of 57 publications since the synonymization include typing), thereby conflating differences between the 2 lineages. Seventeen years after the synonymization, differences between the pathology and host range of the 2 lineages are insufficiently understood. The change also resulted in discovery of either M- type or O-type ‘*M. refringens*’ requiring reporting to WOAH and to the European Commission under Directive EC/2006/88, even in the absence of mortalities (European Food Safety, 2008). The result of the synonymization was an enlargement of the apparent host range of *M. refringens*, which not only included *O. edulis* but also *My. edulis* and *My. galloprovincialis*. As a consequence, several sites in Northern Europe, where *M. refringens* type M (i.e. *M. pararefringens*) was detected were considered positive for *M. refringens*, despite no *Marteilia* detection by histology in oysters (Laing et al. 2014). Disputing the synonymization, a phylogenetic study carried out by Kerr et al. (2018) sequenced almost full length rRNA gene assemblies from O- and M-type *M. refringens*, and determined that the 2 lineages within *M. refringens* were robustly distinguishable at species level, with the full ITS1 sequence being a reliable marker to discriminate between the 2 species. As such, they were formally redefined as separate species: *M. refringens* (previously O-type) and *M. pararefringens* (previously M-type). As there was no material available for *M. maurini* to test the hypothesis that it was synonymous with M-type (and thus synonymous with *M. pararefringens*). To prevent confusion, for the present study we herein adopt the same terminology as in Bøgwald et al. (2022), referring to *M. refringens* as described in Kerr et al. (2018) as *M. refringens sensu stricto*, and *M. refringens* in publications where it is not possible to determine the genotype as *M. refringens sensu lato. Marteilia refringens sensu stricto* and *M. pararefringens* have overlapping but distinct host and geographical distributions; however, data on confirmed infections in potential host species (supported by both histopathology and molecular data) remain sparse (Le Roux et al. 2001; Audemard et al. 2002; López-Flores et al. 2004, 2008a, 2008b; Kerr et al. 2018; Bøgwald et al. 2024).

### Molecular markers for species demarcation

As there is no generally accepted basis for delimiting species in parasitic protists (or for microeukaryotes in general), the definition of species boundaries is a highly debated topic (Boenigk et al. 2012). However, as molecular techniques for the detection of parasites are progressing (i.e. metagenomes, 18S amplicon-based studies, etc.), discovery and characterization of organisms closely related to known parasites is likely to become more common. An understanding of how these closely related parasites have evolved is important to be able to correctly describe them taxonomically. Many molecular markers, particularly within Ascetosporea, target the 18S and ITS1 regions of the rRNA array. However, few sequences exist for the remainder of the array, leaving sequence variability in these organisms poorly understood. The present study applies a method developed by Hooper et al. (2023) to sequence the rRNA array in its entirety in 2 overlapping amplicons, enabling rapid generation of sequence data.

A criticism of the phylogenetic study carried out by Kerr et al. (2018), which formally classified *M. refringens sensu stricto* and *M. pararefringens* as separate species, was the small number of samples that the analysis was performed on: almost complete arrays were produced for only 2 independent *M. pararefringens* samples (9,756–9,777 bp) and 1 *M. refringens sensu stricto* sample (8,559 bp); incomplete arrays were produced for 3 independent *M. refringens* samples (4,031–5,910 bp) and 1 *M. pararefringens* sample (6,811 bp). The incompleteness of the arrays was the result of the primer walking method used to generate them – gaps were present where primer sets failed to amplify regions of the array. Due to the small sample size used and the incompleteness of the arrays, it has not previously been possible to determine consistently different nucleotide differences between *M. refringens sensu stricto* and *M. pararefringens* outside of the ITS1 region. The inferred phylogenetic distinctiveness of the 2 species was based on a very small sample set, and the inference may have been undermined by sequence variation in additional samples.

In this study, we sequenced full rRNA arrays, 18S (small ribosomal subunit) – ITS1 (first internal transcribed spacer) – 5.8S (component of the large ribosome subunit) – ITS2 (second internal transcribed spacer) – 28S (component of the large ribosome subunit) – IGS (intergenic spacer containing the NTS (non-transcribed spacer) and ETS (external transcribed spacer)), for a larger number of *M. refringens sensu stricto* and *M. pararefringens* samples from France, Greece, Italy, Norway, Spain and the United Kingdom using the method outlined in Hooper et al. (2023). Sequence and phylogenetic analyses were carried out on full rRNA arrays, and the most discriminating regions of the rRNA array, of *M. refringens sensu stricto* and *M. pararefringens*, from all available samples covering the known hosts and covering the full documented European geographical range of these parasites. Results of these analyses were used to determine new – and confirm the appropriateness of current – markers to discriminate each species and inform the development of improved diagnostic assays.

## Materials and methods

### Samples

Samples that had previously been shown to be PCR positive for *M. refringens sensu stricto* or *M. pararefringens* were used in this study and are outlined in Supplementary Table 1. This sample set was as comprehensive as possible, covering as much of the host range and geographical distribution of the 2 *Marteilia* species held by the WOAH reference laboratory for marteiliosis and contributed by EU partners.

### PCR to generate long rRNA amplicons

PCR reactions to generate long amplicons were carried out in triplicate in 25 µl volume reactions comprising 1 × PrimerSTAR GXL Buffer (Takara Bio, Shiga, Japan), 0.2 µM dNTP mixture (Takara Bio), 0.4 µM of each primer and 1.25 U PrimeSTAR GXL DNA polymerase (Takara Bio). Cycling conditions for all long amplicon PCRs were carried out by initial denaturing at 95°C for 5 min, followed by 40 cycles of 95°C for 45 s, a variable annealing temperature (described below) for 45 s and 68°C for 150 s; and final extension of 10 min at 68°C.

**Figure 1.**
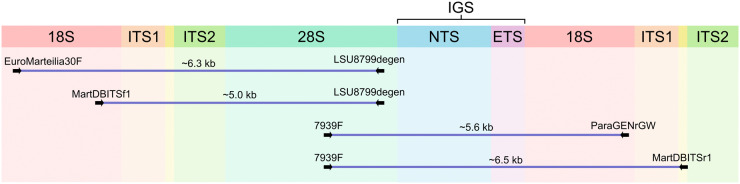
Schematic illustrating the position of primers used for long-range PCR on the of the ribosomal RNA array and the approximate length of amplicons generated by them. 18S – gene for the small subunit of the ribosome, 28S and 5.8S – genes of the components of the large subunit of the ribosome, ITS1 - first internal transcribed spacer, ITS2 - second internal transcribed spacer, IGS - intergenic spacer, NTS - non-transcribed spacer of IGS, ETS - external transcribed spacer of the IGS.

Amplicons covering 18S-ITS1-5.8S-ITS2-28S or ITS1-5.8S-ITS2-28S were amplified with EuroMarteilia30F/LSU8799degen with an annealing temperature of 64°C or MartDBITSf1/LSU8799degen with an annealing temperature of 65°C, respectively. Amplicons covering 28S-IGS-18S or 28S-IGS-18S-ITS1 were amplified using 7939 F/ParaGenRGW with an annealing temperature of 64°C or 7939 F/MartDBITSr1 with an annealing temperature of 63°C. The orientation of primers and the regions of the rRNA array they amplify are detailed in Figure 1, and all primer sequences are given in Table 1.

**Table 1.** Primer sequences, their position of the primer on the rRNA and the direction in which they amplify

Primer name	Sequence (5ʹ–3ʹ)	Location on array	Direction	Usage	Reference
EuroMarteilia30F	CTAGCGTTCGAGACTAAGCC	18S	Forward	Long-range PCR	(Skujina et al. 2022)
ParaGENrGW	GTGTACAAAGGRCAGGGACT	18S	Reverse	Long-range PCR	(Ward et al. 2016)
LSU8799degen	CGAAGRATCAAAAAGCRVCGTC	28S	Reverse	Long-range PCR	(Hooper et al. 2023)
7939 F	GTGACGYGCAYGARTGGADYAAC	28S	Forward	Long-range PCR	(Hooper et al. 2023)
MartDBITSf1	CTCGTGGAGCGGGTCTACCG	18S	Forward	Long-range and first round ITS1 PCR	(Kerr et al. 2018)
MartDBITSr1	TATCACGCCGCTGAATGCTTTCG	5.8S	Reverse	Long-range and first round ITS1 PCR	(Kerr et al. 2018)
MartDBITSf2	TACGCCGTGCCGAGAGGTAC	18S	Forward	Nested ITS1 PCR	(Kerr et al. 2018)
MartDBITSr2	GCGTCCTTCTACGGCTCACGATC	5.8S	Reverse	Nested ITS1 PCR	(Kerr et al. 2018)
INT2RC	GACGAGCGCGAGTGATAGAC	ETS	Forward	First round ETS PCR	Reverse complement of INT2 from López-Flores et al. (2004)
18S	TTGCGCGCCGAAGCACGCTA	18S	Reverse	First round ETS PCR	(López-Flores et al. 2004)
ETSfN	TCTAGCTACTCGAACAACCG	ETS	Forward	Nested ETS PCR	This study
EuromartRC	GGCTTAGTCTCGAACGCTAG	18S	Reverse	Nested ETS PCR	Reverse complement of EuroMarteilia30F from Skujina et al. (2022)

### Sequencing using Illumina and nanopore technologies

Amplicons were cleaned using AMPure XP beads following the manufacturer’s protocol using a 1 × bead volume. All long amplicons were prepared for sequencing on an Illumina MiSeq (Illumina, California, USA) using a Nextera XT DNA Library Preparation Kit (Illumina), following the manufacturer’s protocol, but using half volume reactions. Libraries were sequenced on v2 Nano flow cells (500-cycle) (Illumina) with 2 × 250 bp read length.

A subset of amplicons covering 28S-IGS-18S and 28S-IGS-18S-ITS1 were prepared for Nanopore sequencing using the PCR Barcoding Kit (Oxford Nanopore Technologies, Oxford, UK) and sequenced on Flongle Flow Cells (12 samples per flow cell) (Oxford Nanopore Technologies) using a MinIon Mk1C device (Oxford Nanopore Technologies). Base calling was completed in real-time on the Mk1C device using Guppy (Oxford Nanopore Technologies).

### Sequence analysis of Illumina and nanopore reads

For amplicons sequenced using Illumina MiSeq, raw paired-end sequence reads were trimmed to remove adaptor and low-quality sequences using Trimmomatic v0.39 (using a sliding window of 4, minimum quality of 15, leading and trailing values of 3 and a minimum length of 50 bases; Bolger et al. (2014)). The quality of trimmed and filtered reads was assessed using FastQC v0.11.8 (default parameters; https://www.bioinformatics.babraham.ac.uk/projects/fastqc/) prior to assembly using SPAdes v3.13.1 (in – meta mode, using kmer sizes of 21, 33, 55, 77, 88 and 127; Prjibelski et al. (2020)).

Nanopore-generated raw reads were trimmed, corrected and a consensus generated using Canu v2.1 (default parameters with a genome size of 0.0065 m; Koren et al. (2017)). Trimmed and filtered Illumina paired reads were concatenated using a python script (https://github.com/isovic/racon/blob/master/scripts/racon_preprocess.py) and mapped to the consensus Nanopore contig using minimap2 v2.17-r941 (using -x sr; Li (2018)). Using the mapped reads, the Nanopore-generated contig was polished once using racon v1.4.13 (using default parameters; Vaser et al. (2017)). Nanopore reads were only required to determine the sequence of the non-transcribed spacer (NTS) of the rRNA array, which contains tandem repeats (Hooper et al. 2023). The NTS region was determined not to be useful for this study due to the variation in tandem repeat number within a given sample, so Illumina sequencing alone was used to obtain ETS-28S sequence data due to its higher accuracy rate compared to Nanopore-based sequencing.

Paired reads from each sample were mapped to their respective consensus sequence using BWA-MEM v0.7.17 (Li and Durbin, 2009) and SAMtools v1.9 (Li et al. 2009) with default parameters. The output from BWA-MEM was visualized with Integrative Genomics Viewer (IGV) v2.5.2 (Robinson et al. 2011) and the presence of any single-nucleotide polymorphisms (SNPs) within an individual sample was assessed with, any SNPs present at ≥5% considered. Coverage, assembly quality and accuracy, and using QualiMap v2.2.2 (García-Alcalde et al. 2012).

### PCR to generate short amplicons covering diagnostic regions

PCR reactions to generate shorter amplicons covering the most variable regions of the rRNA array (ITS1 and ETS) were carried out in 20 µl reactions containing 1 μl DNA template, 1 × Green GoTaq® Flexi Buffer (Promega, Wisconsin, USA), 2.5 mM MgCl_2_ (Promega), 0.4 mM dNTP mix (Bioline, London, UK), 0.5 µM of each primer, 10 µg Bovine Serum Albumin (BSA) (New England Biolabs, Massachusetts, USA) and 0.5 u GoTaq® G2 DNA Polymerase (Promega). For ITS1, the nested primer set MartDBITSf1/MartDBITSr1 and MartDBITSf2/MartDBITSr2 was used to amplify a 1,034-bp fragment (Kerr et al. 2018). For ETS, the nested primer set INT2_RC/18S and ETS_FN/EuroMartRC was used to amplify a 1,132-bp fragment. Primer ‘18S’ was published by López-Flores et al. (2004), and ‘INT2RC’ and ‘EuroMartRC’ are the reverse complement of ‘INT2’ and ‘EuroMarteilia30F’ primers published by López-Flores et al. (2004) and Skujina et al. (2022), respectively. Primers were checked for specificity to *Marteilia* using Primer-BLAST (Ye et al. 2012).

Amplicons covering full-length ITS1 generated using MartDBITSf1/MartDBITSr1 and MartDBITSf2/ MartDBITSr2 primers were amplified by an initial denaturing at 94°C for 5 min, followed by 35 cycles of 94°C for 1 min, 65°C for 1 min and 72°C for 1 min; and a final extension of 7 min at 72°C. Amplicons covering a variable region of the ETS were generated using INT2RC/18S primers by an initial denaturing at 95°C for 5 minutes, followed by 35 cycles of 95°C for 1 minute, 63°C for 1 minute and 72°C for 1 minute; and a final extension of 10 minutes at 72°C. The nested amplicon produced by ETSfN/EuroMartRC was amplified as above but with an annealing temperature of 60°C. All primer sequences are detailed in Table 1.

### Sequencing and analysis of ITS1 and ETS amplicons

Amplicons produced by ITS1 and ETS nested PCRs were cleaned using AMPure XP beads following the manufacturer’s protocol using a 1 × bead volume. Cleaned amplicons were sequenced bidirectionally using the nested amplification primers using Eurofins PlateSeq or Eurofins TubeSeq Service (Eurofins Genomics, Wolverhampton, UK).

### Cloning of ITS1 amplicons

When the presence of multiple sequence types was indicated by mixed chromatograms, the first round and nested PCRs were repeated with a proof-reading polymerase, followed by cloning to ensure high sequence accuracy. PCR products that were cloned are indicated with (*) in Table S1. Each 25 µl PCR contained 2 × NEBNext® Ultra™ II Q5® Master Mix (New England Biolabs), 0.5 µM of each primer and 1.25 µl of template DNA. Full ITS1 was amplified by an initial denaturing at 98°C for 30 s, followed by 35 cycles of 98°C for 10 s, 65°C for 30 s and 68°C for 45 s; and a final extension of 2 min at 68°C. PCR products were cleaned using AMPure XP beads following the manufacturer’s protocol using a 1 × bead volume. Cleaned amplicons were heat shocked into plasmids using CloneJet™ PCR cloning kit (Thermo Fisher Scientific, Massachusetts, USA), and DH10B competent cells (Thermo Fisher Scientific) were transformed following the manufacturer’s protocol for blunt ended PCR products. Twenty-two colonies from each sample were taken forward for colony PCR using the MartDBITSf2 and MartDBITSr2 primer set, as above, cleaned using 0.8 × AMPure XP beads, and sequenced bidirectionally using Eurofins PlateSeq service (Eurofins Genomics).

### Phylogenetics

Given the inherent difficulties achieving convergence using a Maximum Likelihood (ML) approach to tree construction from multiple sequence alignments with low-information data (Beerli, 2006) (e.g. regions of the rRNA array that appear to have low mutation rates), a Bayesian approach was taken to construct consensus trees for full transcribed rRNA arrays and the most informative regions of the rRNA array. A Bayesian consensus tree was constructed from ETS-28S rRNA array alignments, produced using MAFFT v7.0 (L-INS-I algorithm; Katoh and Standley (2013)), with MrBayes v3.2.7 (Ronquist et al. 2012) on the CIPRES Science Gateway (Miller et al. 2010). The tree was constructed using 2 separate MC^3^ runs, carried out for 2 million generations using 1 cold and 3 hot chains. The first 500,000 generations were discarded as burn-in, and trees were sampled every 1000 generations. Robust clade distinction was based on maximal support of branching events (posterior probabilities of 1).

For the regions of the rRNA that appeared to be most consistently different between *M. pararefringens* and *M. refringens sensu stricto* (ITS1 and ETS), all publicly available sequences were downloaded from NCBI and aligned against the sequences generated in this study with MAFFT, as above. Ambiguous sequences were removed and Bayesian consensus trees for these regions were constructed as above.

### Coalescent-based species delimitation

Species boundaries within the *Marteilia* clade were inferred using 2 methods. First, a Bayesian Poisson Tree Process (bPTP) model was used to delimit species on the rooted Bayesian consensus tree constructed from a *Marteilia* ETS-28S rRNA array multiple sequence alignment. bPTP analysis was performed on the bPTP web server (Zhang et al. 2013), modelled using 100,000 MCMC generations, a thinning factor of 100, a burn-in of 0.1, and the seed set to 123. Convergence was checked by visually examining the likelihood trace plot. The values corresponding to partitions found by a simple heuristic search were added to the branches of the Bayesian consensus tree.

Secondly, BPP (Flouri et al. 2018), a Bayesian Markov chain Monte Carlo (MCMC) program, was used to determine the posterior probability of inferred species by applying the multispecies coalescent model. The topology of the Bayesian consensus tree generated from *Marteilia* ETS-28S rRNA array multiple sequence alignment was used as a guide tree for species delimitation, and the inferred 5 *Marteilia* species from bPTP analysis were used to assign species to each full array for the Imap input file. To evaluate the influence of prior distributions for ancestral population sizes (θ) and the root age (τ_0_), 3 different combinations of priors were used for the analysis. Priors are assigned a gamma G(α, β) distribution and were applied to the full array to account for:

- large ancestral population sizes and deep divergence between species, *θ* *∼* G(1, 10) and τ_0_ ∼ G(1, 10);
- large ancestral population sizes and shallow divergence between species, *θ* *∼* G(1, 10) and τ_0_ ∼ G(2, 2000);
- small ancestral population sizes and shallow divergence between species, *θ* *∼* G(2, 2000) and τ_0_ ∼ G(2, 2000).

The analyses were performed with the following parameters: rjMCMC algorithm = 0, fine-tuning parameter ɛ = 15, usedata = 1, cleandata = 0 and speciesmodelprior = 1. The rjMCMC analyses were run for 50,000 generations (sampling interval of 5) with a burn-in period of 5,000. Each analysis was run in duplicate to ensure consistent results across analyses initiated with different starting seeds.

## Results

### Assembly of arrays and multiple sequence analysis

Full transcribed arrays (ETS-28S; approximately 8,380 bp) were assembled from 9 independent samples for *M. refringens sensu stricto* (*M. refringens* as described in Kerr et al. (2018)) and 28 independent samples for *M. pararefringens* by merging the overlapping fragments for each sample to produce 1 contig. The non-transcribed spacer (NTS) region of the intergenic spacer (IGS) was excluded from analysis due to its tandem repeat structure, making it an unsuitable region for determining differences between the 2 *Marteilia* species. The full transcribed regions of the rRNA arrays generated in this study were deposited to GenBank under accession numbers PP549144-80, with details of the host, location from which they were amplified and average contig coverage given in Supplementary Table 1.

A total of 20 consistent nucleotide differences (differences seen in all samples of each *Marteilia* species) were observed across the 8,410 nucleotide positions of transcribed rRNA (ETS-28S) alignments of *M. refringens sensu stricto* and *M. pararefringens*, including the 7 complete or near complete rRNA arrays generated by Kerr et al. (2018). The majority of these differences were within the ETS (11 nucleotide differences over 1,508 bp) and ITS1 (6 nucleotide differences over 690 bp). The remaining nucleotide differences were located in ITS2 (1 nucleotide difference) and the 5ʹ end of the 28S (2 nucleotide differences). *Marteilia refringens sensu stricto* and *M. pararefringens* had 100% sequence similarity in 18S (except samples from Norway, described below) and 5.8S regions of the rRNA array.

**Figure 2.**
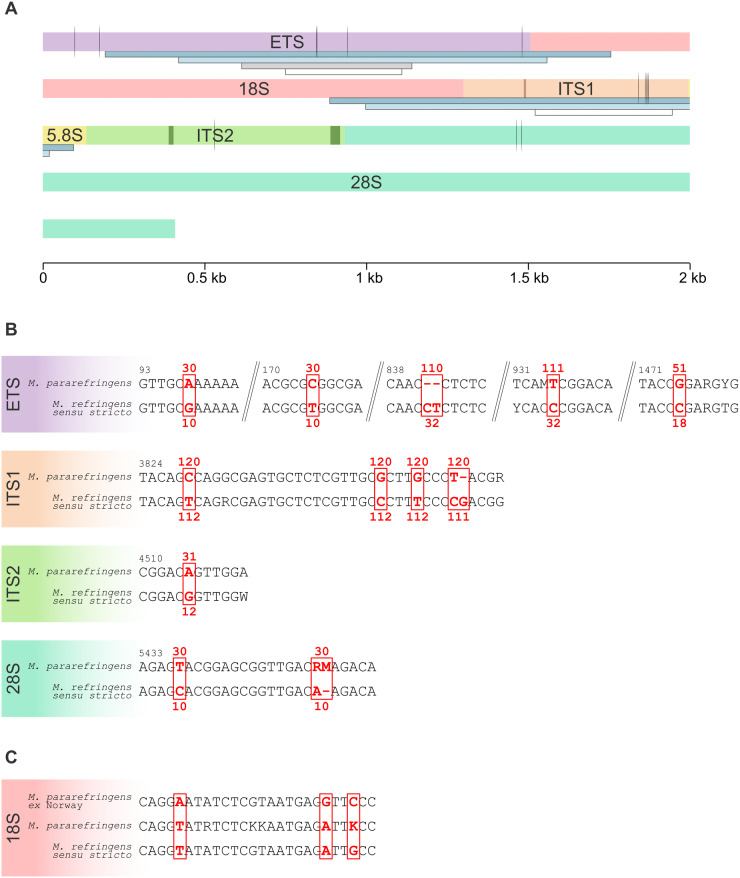
Consistently different nucleotide differences between *Marteilia pararefringens* and *M. refringens sensu stricto.* (A) Schematic representation of full transcribed regions of the ribosomal RNA (rRNA) arrays for *M. pararefringens* and *M. refringens sensu stricto.* Vertical black lines represent consistent differences between species. Darker blocks in ITS1 and ITS2 represent tandem repeat regions. Grey and white horizontal blocks in under the ETS represent regions amplified by López-Flores et al. (2004) nested primer set and dark and light blue blocks under the 18S and ITS1 represent regions amplified by Kerr et al. (2018) nested primer set. Dark and light blue horizontal blocks under the ETS and 18S represent the region amplified by nested primers designed in this study, and the white horizontal block under ITS1 represents the region amplified by the Le Roux et al. (2001) pr4/pr5 primer set. (B) Details of the consistently different nucleotide positions depicted in A. Red bases are invariant within each species, with the position of these bases relative to sample 18/35/12 (accession number PP549162). Numbers above and below the highlighted bases depict the number of samples for each species that were available for these regions to determine the differences. (C) Consistent nucleotide differences in the 18S gene between *M. pararefringens* amplified from samples from Norway compared to all other *M. pararefringens,* and *M. refringens sensu stricto* samples.

The ITS2 region of the rRNA had only 1 consistent nucleotide difference between species across its 800-bp length. Mapping Illumina reads to the consensus contigs obtained from SPAdes assembly revealed that ITS2 in *M. pararefringens* had regions containing indels (shown as dark green blocks in Figure 2A), which occurred in short tandem repeat regions. For example, within sample 2006_031_11, 1, 2 or 3 copies of ACA (ungapped position 4,376 bp on the rRNA) were present. Within 28S, 2 short regions (∼50 bp) at the 3ʹ end of the gene appeared to have bases that were variable within a sample. This variability was consistent across both *M. pararefringens* and *M. refringens sensu stricto* samples, with the majority of ambiguity occurring as T to G base changes. A shorter region of variability (∼20 bp) was also observed at the 5ʹ end of the 18S gene. Within this region, there were no consistent nucleotide differences between *M. pararefringens* and *M. refringens sensu stricto*; however, *M. pararefringens* from Norway showed 3 consistently different nucleotide differences with respect to all other *M. pararefringens* sequences, these differences are shown in Figure 2C. Across the remainder of the array, there were 3 other nucleotide positions, 2 in ETS and 1 in ITS2, which were consistently different in samples from Norway than other *M. pararefringens* sequences.

### Amplification of the most discriminating regions of the rRNA array

To confirm the differences in ETS and ITS1 over a larger sample set, amplification and Sanger sequencing using nested PCR assays was carried out. ITS1 and ETS sequences generated in this study were deposited to GenBank under accessions PP544135-53 and PP549181-98, respectively, with details of the host and location from which they were amplified given in Supplementary Table 1.

#### Internal transcribed spacer 1

As the MartDBITSf1/MartDBITSr1 and MartDBITSf2/MartDBITSr2 ITS1 nested primer set (Kerr et al. 2018) amplified all consistently different nucleotides in the ITS1 region between the 2 *Marteilia* species identified by long-range PCR and sequencing, this primer set was applied to all available samples. The resulting sequences were aligned with the ITS1 region of the full-length arrays and all available ITS1 sequences on GenBank, totalling 120 *M. pararefringens* amplified from 6 different species, and 112 *M. refringens sensu stricto* sequences amplified from 3 different species (Table 2). Identical sequences were collapsed into sequence types; the sequences that constitute each type are outlined in Supplementary Table 2. Four ITS1 sequences were removed from the analysis: DQ426583, DQ426587 and DQ426596 due to sequencing reads appearing to be chimeric clones (a single DNA sequence originating from the joining of multiple DNA sequences caused by dissociation of polymerases from the template during sequencing). A proportion of the sequence matched an *M. pararefringens* clone, and the remaining proportion matched the *M. refringens sensu stricto* clone generated from the same sample. MH304865 was removed as the chromatogram showed mixed sequence types.

**Table 2.** Species from which *M. pararefringens* and *M. refringens sensu stricto* were amplified from for ITS1 and ETS phylogenetic analysis. Data include sequences from NCBI and those generated in this study. Spores *ex Ostrea edulis/Mytilus galloprovincialis* refer to a sequence derived from *Marteilia* spores purified from a pool of tissues from both species

	Proportion of sequences per species (%)			
	ITS1		ETS	
	*Marteilia*	*Marteilia refringens*	*Marteilia*	*Marteilia refringens*
Species from which *Marteilia* sequences were derived	*pararefringens n* = 121	*sensu stricto n* = 112	*pararefringens n* = 111	*sensu stricto n* = 32
*Mytilus edulis* 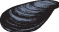	28.1	0.0	33.3	0.0
*Ostrea edulis* 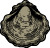	9.9	55.4	0.9	75.0
*Mytilus galloprovincialis* 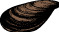	47.1	38.4	31.5	3.1
Plankton 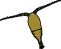	12.4	0.0	25.2	0.0
*Ostrea stentina* 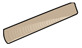	1.7	6.3	0.0	0.0
*Crassostrea madrasensis* 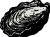	0.8	0.0	0.0	0.0
*Xenostrobus secures* 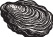	0.0	0.0	4.5	0.0
*Solen marginatus* 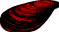	0.0	0.0	4.5	0.0
*Crassostrea corteziensis* 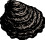	0.0	0.0	0.0	9.4
*Magallana gigas* 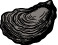	0.0	0.0	0.0	6.3
*Chamelea gallina* 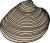	0.0	0.0	0.0	3.1
Spores ex. *Ostrea edulis/Mytilus galloprovincialis* 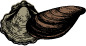	0.0	0.0	0.0	3.1

#### External transcribed spacer

Primers were designed to amplify 1,577 bp (single PCR) or 1,132 bp (nested PCR) of the ETS, producing an amplicon that covered most of the potential nucleotide difference between ETS regions of *M. refringens sensu stricto* and *M. pararefringens* suggested as species-specific markers by scrutiny of full-length rRNA arrays. The regions amplified by the single round and nested PCRs are shown as dark and light blue horizontal blocks on Figure 2A. Two differences at the 5ʹ end of the ETS could not be amplified due to low GC content and homopolymers upstream of this site, making it unsuitable for primer design. The primer set was applied to all available samples and the resulting sequences were aligned with the ETS amplicon region of the full-length arrays and all available ETS sequences on GenBank, totalling 111 *M. pararefringens* amplified from 6 different species, and 32 *M. refringens sensu stricto* sequences amplified from 6 different species (Table 2). Identical sequences were collapsed into sequence types; the sequences that constitute each type are outlined in Supplementary Table 3. Sequencing additional samples and comparison to ETS sequences in GenBank revealed that 5-nucleotide differences initially thought to be unique to each species in the full-length arrays were not unique with a greater sample number.

Consistently different nucleotide differences in the larger sample set are shown in Figure 2A–B. Three publicly available ETS sequences were removed from further analysis: accessions AJ629374-5 were removed due to there being degenerative bases present in clonal sequences, and MH304860, as revisiting the chromogram revealed mixed sequence types. Confirmation and/or disproof of the consistently different nucleotides observed in the limited number of full-length arrays by Sanger sequencing of shorter regions resulted in a total of 15 consistently different nucleotide positions across the full array between the 2 species of *Marteilia*.

### Phylogenetic analysis of full rRNA arrays

**Figure 3.**
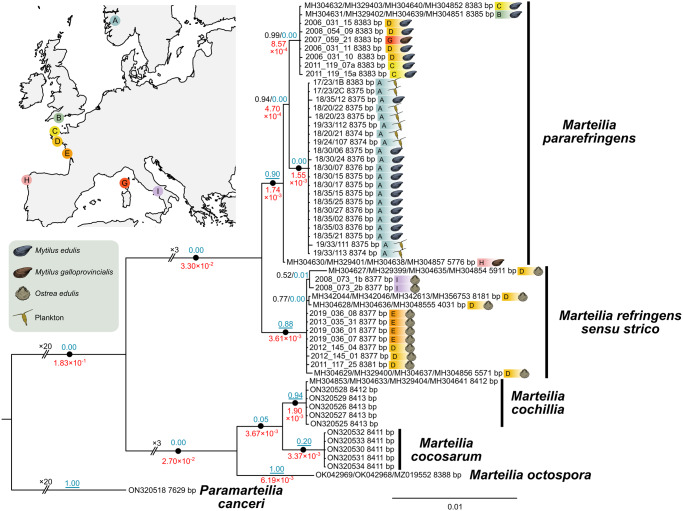
Bayesian consensus tree constructed from the full transcribed region (ETS-28S) of the rRNA array (8,860 nucleotide positions) for *Marteilia refringens sensu stricto* (*n* = 13) and *M. pararefringens* (*n* = 30), *M. cochillia* (*n* = 5), *M. cocosarum* (*n* = 5) and *M. octospora* (*n* = 1). Location of sampling and host species are denoted adjacent to each branch. Posterior probabilities to two significant figures are displayed on the branches in black text, with maximal support values (1.0) shown as black circles. Branch lengths are shown on key branches in red text. The tree is rooted to *Paramarteilia canceri*. Posterior probability values from bPTP analysis for all branching events are shown in blue text on branches. The posterior probabilities of likely speciation events suggested by the bPTP analysis are underlined.

A Bayesian consensus demonstrated maximal support (posterior probabilities of 1) for *M. refringens sensu stricto* and *M. pararefringens* clades. The tree, generated from 13 *M. refringens sensu stricto* and 30 *M. pararefringens* ETS-28S rRNA arrays (8,860 nucleotide positions), demonstrated mutually exclusive, monophyletic clades for both species (Figure 3). All other species of *Marteilia*, where full rRNA arrays were available, branched with maximal support for each species, with similar phylogenetic distances between *M. refringens sensu stricto* and *M. pararefringens* as between other closely related *Marteilia* species (*M. cochillia* and *M. cocosarum*). One long *M. pararefringens* sequence (accession numbers MH304630, MH329401, MH304638 and MH304857) branched separately from other *M. pararefringens* sequences, although still with maximal support with all other *M. pararefringens* sequences. However, this rRNA assembly is partial – lacking the informative region of ITS1 – which is likely to explain this branching pattern. Within the *M. pararefringens* clade, samples from Norway branched separately from other *M. pararefringens* samples, again with maximal support.

### Phylogenetic analyses of ITS1 and ETS regions

**Figure 4.**
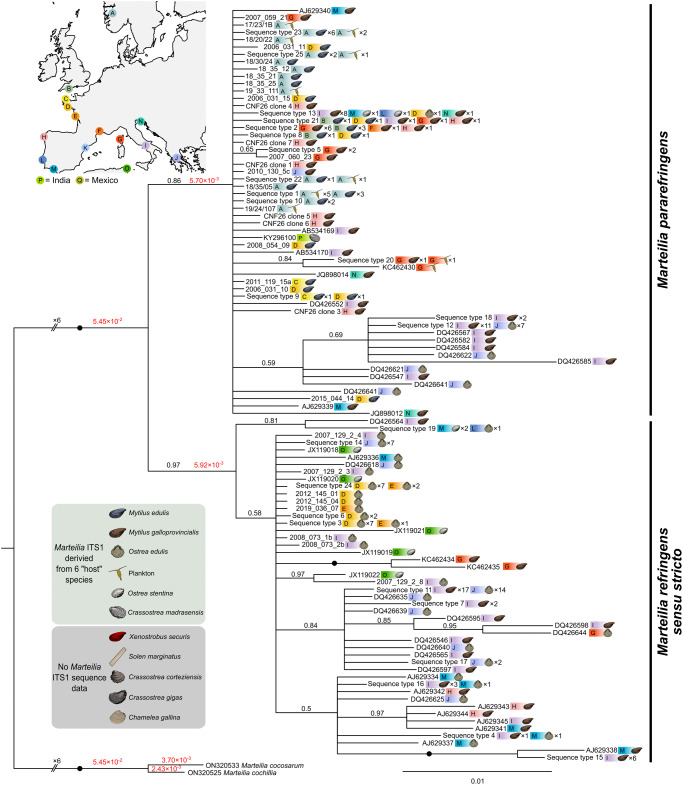
Bayesian consensus tree constructed from full ITS1 sequences (945 nucleotide positions) for *Marteilia refringens sensu stricto* (*n* = 112) and *M. pararefringens* (*n* = 121). Location of sampling, host species and number of individual samples the sequence type was detected in is denoted adjacent to each branch. Posterior probabilities to two significant figures are displayed on the branches in black text, with maximal support values (1.0) shown as black circles. Branch lengths are shown on key branches in red text. Tree is rooted to *M. cochillia/M. cocosarum*.

A Bayesian tree, constructed from all full-length ITS1 sequences generated in this study and ITS1 sequences available on GenBank, produced a highly supported *M. refringens sensu stricto* clade (0.97 posterior probability), and a moderately supported (0.86 posterior probability) *M. pararefringens* clade (Figure 4). Unlike the tree generated from full-length transcribed rRNA arrays, *M. pararefringens* amplified from Norway did not branch separately from other *M. pararefringens* sequences.

**Figure 5.**
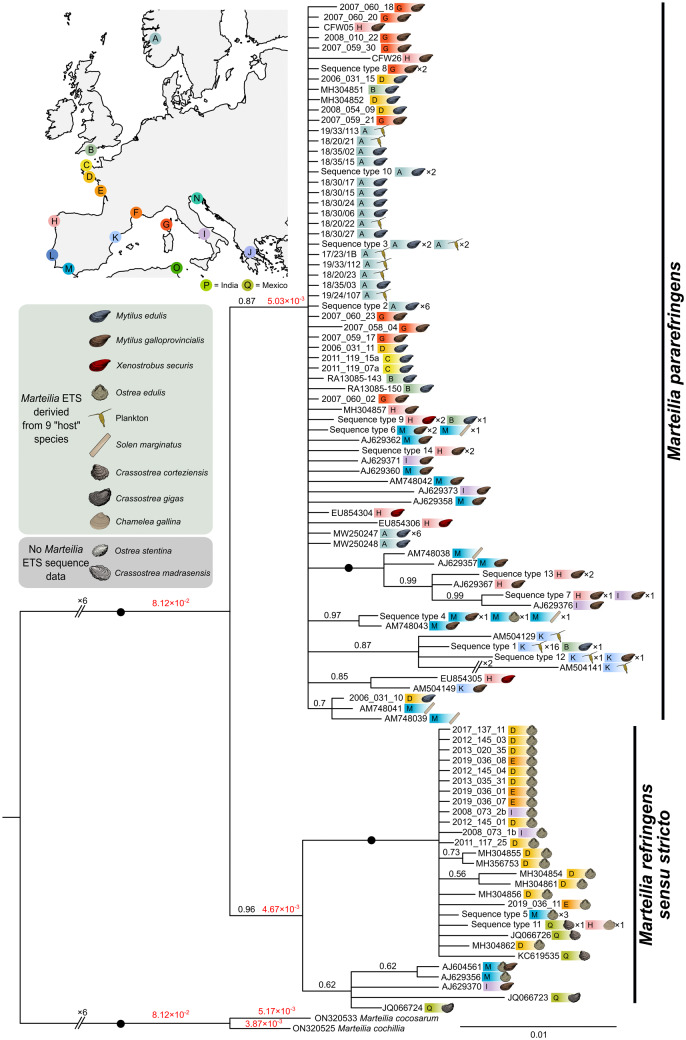
Bayesian consensus tree constructed from long ETS sequences (1,043 nucleotide positions) for *M. refringens sensu stricto* (*n* = 32) and *M. pararefringens* (*n* = 111). Location of sampling and host species is denoted adjacent to each branch. Posterior probabilities to two significant figures are displayed on the branches in black text, with maximal support values (1.0) shown as black circles. Branch lengths are shown on key branches in red text. Tree is rooted to *M. cochillia/M. cocosarum.*

A Bayesian consensus tree constructed from long ETS sequences (1,043 nucleotide positions) generated in this study, and ETS sequences available on GenBank, for *M. refringens sensu stricto* (*n* = 32) and *M. pararefringens* (*n* = 111) produced a tree with similarly moderate support values for each clade as the ITS1 tree, with both species forming mutually exclusive, monophyletic clades (Figure 5).

Trees based on the shorter regions amplified by primers recommended in the Aquatic Animal Health Manual (Woah, 2023) (originally published by Le Roux et al. (2001), Figure 2A) were also constructed. A tree based on short ITS1 sequences produced mutually exclusive monophyletic clades for both *M. pararefringens* and *M. refringens sensu stricto* but had no support (0.51 posterior probability) for the *M. refringens sensu stricto* clade (Supplementary Figure 1A). Trees constructed from short ETS amplicon regions produced trees in which *M. pararefringens* was paraphyletic with respect to *M. refringens sensu stricto* (Supplementary Figure 1B-C), with high support for the *M. refringens sensu stricto* clade. In both ITS1 and ETS regions, longer amplicon regions produced trees that had higher support for each species’ clade than their shorter counterparts.

### Coalescent-based species delimitation

bPTP inferred 5 *Marteilia* species (*M. refringens sensu stricto, M. pararefringens, M. cochillia, M. cocosarum and M. octospora*) from analysis of the *Paramarteilia canceri* rooted ETS-28S Bayesian consensus tree (Figure 3). Moderate to high posterior probability values were seen for *M. refringens sensu stricto* and *M. pararefringens* (0.88 and 0.90, respectively). Despite samples from Norway branching separately from all other *M. pararefringens* with maximal support on the Bayesian consensus tree, bPTP analysis did not infer this branching pattern as a speciation event (posterior probability = 0.00). Posterior probability values from bPTP analysis for all branching events are shown in blue text on branches in Figure 3, with the posterior probabilities of likely speciation events suggested by the bPTP analysis indicated by an underlined posterior probability.

**Figure 6.**
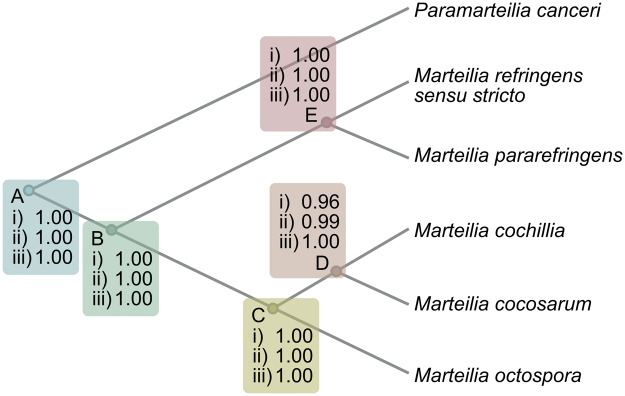
Bayesian species delimitation results for *Marteilia* based on the full transcribed rRNA multiple sequence alignment and assuming a 5-species guide tree rooted to *Paramarteilia canceri.* The speciation probabilities are provided for each node under each combination of priors for θ and τ_0_: (i) *θ ∼* G(1, 10) and τ_0_ ∼ G(1, 10); (ii) *θ ∼* G(1, 10) and τ_0_ ∼ G(2, 2000); and (iii) *θ ∼* G(2, 2000) and τ_0_ ∼ G(2, 2000).

To investigate the validity of the 5 hypothesized *Marteilia* species inferred from bPTP, BPP analyses were performed. Bayesian species delimitation supported the guide tree with speciation probabilities of ≥0.95 on all nodes (Figure 6), a probability that other studies have considered as strong support for a speciation event (Leaché and Fujita, 2010). *M. refringens sensu stricto* and *M. pararefringens* are supported by a speciation probability of 1.00, with different prior distributions for *θ* and τ_0_ not affecting this outcome (node E on Figure 6). Speciation probabilities of 1.00 were consistent for all tested combinations of priors for all nodes except for the *M. cochillia and M. cocosarum* speciation event. Speciation probabilities for *M. cochillia* and *M. cocosarum* increased from 0.96 to 0.99/1.00 using priors that assumed that these 2 species had recently diverged (node D on Figure 6).

### *Inferred host preference of* M. pararefringens *and* M. refringens sensu stricto *based on available sequence data*

Phylogenetic trees in this study were generated using newly generated sequence data and all publicly available sequences. Once generated, the host from which the sequence was amplified was mapped onto the branches of the tree to determine if there were any differences between the 2 *Marteilia* species; the results of this are summarized in Table 2. One of the clearest differences between the 2 *Marteilia* species was that no publicly available *M. refringens sensu stricto* sequences from any part of the rRNA array exist from *My. edulis* or non-bivalve species, although a singular detection of *M. refringens sensu stricto* was reported from *My. edulis* by Le Roux et al. (2001) using RFLP analysis. *M. pararefringens* has been frequently amplified from *My. edulis*, with 28.1% and 33.3% of all *M. pararefringens* ITS1 and ETS sequences amplified from this species.

Due to the low sample numbers for *M. refringens sensu stricto*, the ETS region did not provide enough information to compare potential hosts, so ITS1 was used as a proxy, with 121 sequences classified as *M. pararefringens* and 112 sequences classified as *M. refringens sensu stricto.* Although some *M. pararefringens* sequences were amplified from *O. edulis* (9.9%), a larger percentage of sequences amplified from this species branched with *M. refringens sensu stricto* (55.4%). There did not appear to be a clear *Marteilia* species preference in *My. galloprovincialis* with similar numbers of sequences amplified for each *Marteilia* species: 47.1% *M. pararefringens* and 38.4% *M. refringens sensu stricto.* As several of the sequences deposited on GenBank were generated by sequencing of clones, the exact number of animals that these sequences were derived from is unclear as multiple clones may have originated from the same *Marteilia* amplicon.

## Discussion

In this study, we used high-throughput sequencing to generate full rRNA arrays from 9 independent *Marteilia refringens sensu stricto* (*M. refringens* as described in Kerr et al. (2018)) and 28 independent *M. pararefringens* samples. Bayesian phylogenies generated from full rRNA arrays, and the most variable regions of the rRNA (ITS1 and ETS) produced mutually exclusive monophyletic clades for each species, with high to maximal support for each clade. These phylogenies continue to support the separate species status of *M. refringens sensu stricto* and *M. pararefringens*, as defined in Kerr et al. (2018). The robust definition of the consistent nucleotide difference between *M. refringens sensu stricto* and *M. pararefringens* will allow the study of these parasites in isolation to determine their individual and combined impacts on bivalve culture in affected regions.

### *Genomic differences between* M. refringens sensu stricto *and* M. pararefringens

Two publications in the early 2000s described consistent nucleotide differences observed in small sections of the rRNA array (ITS1 and ETS) between the 2 *Marteilia* species. In ITS1, Le Roux et al. (2001) described 5 nucleotide differences over a 413 bp region, which were confirmed by López-Flores et al. (2004). From the samples analysed in this study, we confirm that these differences are still robust for discriminating between the 2 species in PCR positive samples from 2002 to 2019 (Figure 2B). López-Flores et al. (2004) described 2 differences between the 2 species over a 525 bp region of ETS. Here, we describe 6 consistent nucleotide differences across the whole region. Four of these nucleotide differences were amplified from a larger *M. refringens sensu stricto* and *M. pararefringens* sample set using a novel nested PCR assay. The similarity between *M. refringens sensu stricto* and *M. pararefringens* over the full ETS (99.60%) is higher than that of other closely related but distinct paramyxid species: *Marteilia cochillia* and *M. cocosarum* have 99.03% similarly and *Paramarteilia canceri* and *P. orchestiae* have 97.81% similarity (Hooper et al. 2023). However, when branch lengths are compared (Figure 5), *M. refringens sensu stricto* and *M. pararefringens* appear more distantly related to each other than *M. cocosarum* is to *M. cochillia*, closely related *Marteilia* species that are clearly delimited as separate species due their mode of infection (Skujina et al. 2022).

Due to intragenomic variation, or coamplification of *M. refringens sensu stricto* and *M. pararefringens*, particularly in *My. galloprovincialis*, it has been necessary for some previous studies to clone PCR products to acquire a clean sequence. Many cloned sequences on NCBI have nucleotide differences that were not reproduced, even at low frequencies, in high-throughput or Sanger sequencing in this study. It is possible that these differences are artefacts from PCRs that have used standard *Taq* polymerases, which are prone to errors. These errors are unlikely to be seen when sequencing a PCR product directly, but errors may become more prevalent after further amplification of single ligated PCR products during the cloning process. We excluded 3 ITS1 sequences that were suspected to have sequencing artefacts from cloning from our analysis. To remove any ambiguity in sequence data, we recommend that high-fidelity, proof-reading polymerases should be used for sensitive downstream applications, such as looking at intraspecies variation and coinfections by cloning and Nanopore sequencing.

Preliminary data from Kerr et al. (2018) suggested that 28S and ITS2 may also be useful regions for discriminating between *M. refringens sensu stricto* and *M. pararefringens*; however, analysis of a larger number of samples in this study showed that both of these regions are not suitable as taxonomic markers. The 28S was shown to have only 2 consistently different nucleotides, located within a 100 bp region of the 5ʹ end, with the remainder of the gene being identical in sequence between species. Kerr et al. (2018) suggested that ITS2 may have up to 13 markers to distinguish between the 2 species; however, this was based on only 3 *M. pararefringens* and 2 *M. refringens sensu stricto* sequences. Here, we find that only one of those nucleotide differences can be reliably used based on a larger sample set. We also find that within samples, there are regions of the ITS2 that have intragenomically variable short tandem repeat regions, making it unsuitable as a marker region due to the requirement to clone PCR amplicons to this region to acquire sequence data.

### Intraspecies variation

Short intragenomic variable regions are also seen in the 28S and 18S, as well as the spacer regions of the 2 *Marteilia* species. Within 28S, these regions are located at the 3ʹ end of the gene, and the same variability is observed in both *M. refringens sensu stricto* and *M. pararefringens*, irrespective of the location or species they were amplified from. A short ∼20 bp region of intragenomic variability is also present at the 5ʹ end of the 18S. Unlike in the 28S, this region only appears to be variable in *M. pararefringens* sequences. Interestingly, within this region of variability, *M. pararefringens* samples from Norway contain 3 nucleotide positions that are unique to samples from this geographical region and differentiate them from all other *M. pararefringens* and *M. refringens sensu stricto* sequences. These nucleotide differences were consistent across all samples from Norway, irrespective of the species they were amplified from or the year they were sampled. The differences are reflected in the full transcribed rRNA array (ETS-28S) phylogenetic tree (Figure 3), where samples from Norway branch robustly within the *M. pararefringens* clade, but separately from all other samples, with maximal support for the clade. Despite these differences appearing in a large number of samples from Norway, all samples used to generate full rRNA arrays originated from 1 estuarine pond (‘poll’) on the West coast of Norway. To investigate the geographic extent of this *M. pararefringens* population-level sequence variant in Norway we sequenced *M. pararefringens* positive mussels from 6 other regions in Norway recently found to have *M. pararefringens-*infected mussels (Bøgwald and Mortensen, 2024) and found that these differences were present in all samples, including 2 *M. pararefringens* sequences originating from *My. trossulus* (Bøgwald et al. 2024). Hypotheses about how this sequence variant arose include (but are not limited to) *M. pararefringens* in Norway having undergone a population bottleneck event, or this sequence variant representing a northern European variant of *M. pararefringens* that may be present in other northern European countries but remains undetected due to the absence of substantial *Marteilia-*associated mortality events in mussels and thus limited surveillance.

### Host preference

Previous publications disputed the species status of *M. refringens sensu stricto* and *M. pararefringens* due to the ability of each species to be detected in a wider host range than originally described (Balseiro et al. 2007). *Marteilia refringens sensu stricto* has been amplified from *O. edulis* (Le Roux et al. 2001) and *My. galloprovincialis* (López-Flores et al. 2004). Sporadic detection of *M. refringens sensu stricto* has been seen in *My. edulis* (Le Roux et al. 2001), the clam *Chamelea gallina* (López-Flores et al. 2008b), and the oysters *Ostrea stentina* (Elgharsalli et al. 2013), *Magallana gigas* and *Crassostrea corteziensis* (Grijalva-Chon et al. 2015; Martínez-García et al. 2017). *M. pararefringens* is commonly found in *My. edulis* and *My. galloprovincialis* (Le Roux et al. 2001). Sporadic detection of *M. pararefringens* has been seen in *O. edulis* (Le Roux et al. 2001), the Pacific blue mussel *My. trossulus* (Bøgwald et al. 2024), the clams *Solen marginatus* (López-Flores et al. 2008a) and *Ruditapes decussatus* (Boyer et al. 2013) and the black-pygmy mussel *Xenostrobus securis* (Pascual et al. 2009). *M. refringens sensu lato* (based on detection without or with limited sequence data) has been found in the oysters *Ostrea chilensis* (Grizel et al. 1982)*, O. angasi* (Bougrier et al. 1986)*, O. puelchana* (Pascual et al. 1991), *O. denselamellosa* (Martin, 1993), and *Crassostrea virginica* (Renault et al. 1995). *Marteilia refringens sensu stricto* has not been detected in Northern Europe, but preliminary data suggests that it may be present in the USA (Grijalva-Chon et al. 2015; Kerr et al. 2018). *Marteilia pararefringens* has been detected in both Northern and Southern Europe (Kerr et al. 2018)*. Marteilia refringens sensu lato* and *M. pararefringens* have also been detected in a number of non-bivalve species including copepods, a cnidarian and a nematode (Audemard et al. 2002; Carrasco et al. 2007; Boyer et al. 2013; Arzul et al. 2014; Bøgwald et al. 2022).

In Table 2, we present the largest dataset to date, collated with respect to the species from which each *Marteilia* species was amplified. We emphasize that this table reports amplification of parasite DNA from host samples, but this does not confirm infection. Positive PCR results may be generated by extracellular parasite DNA, particularly in the case of filter-feeding hosts. Furthermore, interpretation of the results in Table 2 must take in account possible misidentification of *Mytilus* species in some studies, or as hybridization between *Mytilus* species is common (Nascimento-Schulze et al. 2023), undetected hybrids may have been present in *Marteilia* detection studies. 38.4% of publicly available *M. refringens sensu stricto* sequences originated from *My. galloprovincialis*; however, only *M. pararefringens was* amplified from mussels in this study. The available sequences are derived from a limited number of studies, mostly from Spain, and using the cloning approach, so may overrepresent the detection of this species in *My. galloprovincialis.* There are also still no confirmed infections (histopathological identification) of a *Mytilus* species with *M. refringens sensu stricto.*

*M. refringens sensu stricto* from *O. edulis* comprised over 50% of all ITS1 sequences, whereas *M. pararefringens* from *O. edulis* only represents 9.9% of ITS1 sequences. However, although *M. pararefringens* has been detected by PCR from *O. edulis*, there are no confirmed reports of infection by histopathology, even – as reported in Kerr et al. (2018) and Bøgwald and Mortensen (2024) – at sites where *M. pararefringens* has been shown to be an abundant parasite of mussels (e.g. Bømlo, Norway). A recent study introduced naïve *O. edulis* to a site with a historically high prevalence of *M. pararefringens*, and despite high confirmed infections of *M. pararefringens* in *My. edulis*, there was no evidence of infection in introduced or native *O. edulis*, despite weak PCR detection in 10 of the 220 screened oysters (Bøgwald et al. 2025).

### *Defining* M. refringens sensu stricto *and* M. pararefringens *as distinct species*

For *M. refringens*, the infraspecific ‘type’ terminology used for the past couple of decades is not taxonomically recognized, creating uncertainty and ambiguity, and should be avoided. The only infraspecific rank recognized by the ICZN that could be considered for *M. refringens sensu stricto* and *M. pararefringens* is subspecies, but this term is most appropriately used to distinguish morphologically distinct and geographically separate, but not reproductively isolated, populations within a species (Mayr, 1982; Monroe, 1982). These criteria are not applicable to *M. refringens sensu stricto* and *M. pararefringens*.

Following the recommendation that the bases for species definition be made on a case-by-case basis, particularly for microeukaryotes for which many existing species concepts are not applicable for a variety of reasons (Boenigk et al. 2012), we assert that the robust phylogenetic mutual exclusivity of *M. refringens sensu stricto* and *M. pararefringens* and their different host preferences demonstrated by the new data and analyses presented in this paper, in combination with their differing (although overlapping) geographical ranges, provide more than sufficient evidence of their valid status as separate species. In addition to this, we performed coalescent-based species delimitation analysis on the complete transcribed region of the rRNA locus to confirm the validity of the 5 *Marteilia* species for which full rRNA arrays exist (*M. refringens sensu stricto, M. pararefringens, M. cochillia, M. cocosarum*, and *M. octospora*), suggested by phylogenetic and bPTP analysis. These analyses provided maximal support for the *M. refringens sensu stricto* and *M. refringens* speciation event across 3 prior distributions that assumed differing ancestral population sizes and divergence depth.

It is worth restating here: there is no consistent relationship between genetic differences between marker genes used for taxonomic discrimination and phenotypic divergence across eukaryotic taxa. The processes underlying sequence evolution in marker genes and those under adaptive selection are not the same, and the relative rates differ depending on the biology and ecology of the taxa involved at any point in time. Different genes/gene regions evolve at different rates in different taxa, and with different correspondences to phenotypic change. Recently, a genome for *M. pararefringens* has been generated (Hiltunen Thorén et al. 2024). When a comparable genome for *M. refringens sensu stricto* is available, further marker regions outside of the rRNA gene may be identified; however, investigation of other marker genes based on the *M. pararefringens* genome was outside of the scope of this study.

*M. refringens sensu stricto* and *M. pararefringens* are certainly closely related, but their degree of genetic (marker gene) divergence is commensurate with that of other species in the same genus, other microeukaryotes (e.g. the ciliate species *Oxytricha granulifera* and *Ox. atypica*, which differ by only 3 nucleotide sites over a 4127 bp region of the 18S to 28S rRNA gene array (Fan et al. 2021)), the dinoflagellates *Gymnodinium baicalense* and *G. corollarium* (Annenkova et al. 2020), cercomonads (Bass et al. 2007), fungi (for which multi-locus species differentiation is recommended as individual markers (e.g. ITS rDNA) may underestimate species diversity; (Matute and Sepúlveda, 2019)), and even some macroorganisms, such as seaweed species in the genus *Fucus* (Leclerc et al. 1998).

## Summary

We provide further robust evidence to support the separate species status of *M. refringens sensu* stricto and *M. pararefringens*: (1) Phylogenies generated from a larger number of complete rRNA arrays than in Kerr et al. (2018), and all available sequences from the regions of the rRNA array with the most consistently different nucleotides between the 2 *Marteilia* species (ITS1), produce mutually exclusive, monophyletic clades for *M. refringens sensu stricto* and *M. pararefringens*; (2) the 2 *Marteilia* species branched with similar phylogenetic distances to other closely related *Marteilia* species i.e. *M. cochillia, M. cocosarum* and *M. octospora*; (3) coalescent-based species delimitation analyses on the complete transcribed the rRNA array confirmed the validity of 5 *Marteilia* species for which full rRNA arrays exist (*M. refringens sensu stricto, M. pararefringens, M. cochillia, M. cocosarum*, and *M. octospora*) and (4) analysis of sequences collated with respect to the species from which each *Marteilia* species was amplified showed a clearer host preference than studies had previously reported – no current sequence data for *M. refringens sensu stricto* has been amplified from *Mytilus edulis*, and *M. pararefringens* is amplified less frequently in *Ostrea edulis* than *M. refringens sensu stricto.* Use of the robust marker region in ITS1 allows clear distinction between the 2 species and should still be used for monitoring and research studies on both parasite species in order to clarify their respective host preferences, pathogenicity and life cycles.

## Supporting information

Hooper et al. supplementary material 1 Hooper et al. supplementary material

Hooper et al. supplementary material 2 Hooper et al. supplementary material

Hooper et al. supplementary material 3 Hooper et al. supplementary material

Hooper et al. supplementary material 4 Hooper et al. supplementary material

## Data Availability

The full transcribed regions of the rRNA arrays generated in this study are deposited to GenBank under accession numbers PP549144-80, and ITS1 and ETS sequences are deposited under accessions PP544135-53 and PP549181-98, respectively.

## Supplementary material

The supplementary material for this article can be found at https://doi.org/10.1017/S0031182025100796.
