# Supplementary figures and images for: Full ribosomal RNA gene arrays confirm Marteilia refringens sensu stricto and Marteilia pararefringens as separate species, and assess the validity of current diagnostic regions
Source: Parasitology. 2025 Sep 8;152(12):1247–62. doi: 10.1017/S0031182025100796 (PMC12921245; doi:10.1017/S0031182025100796)

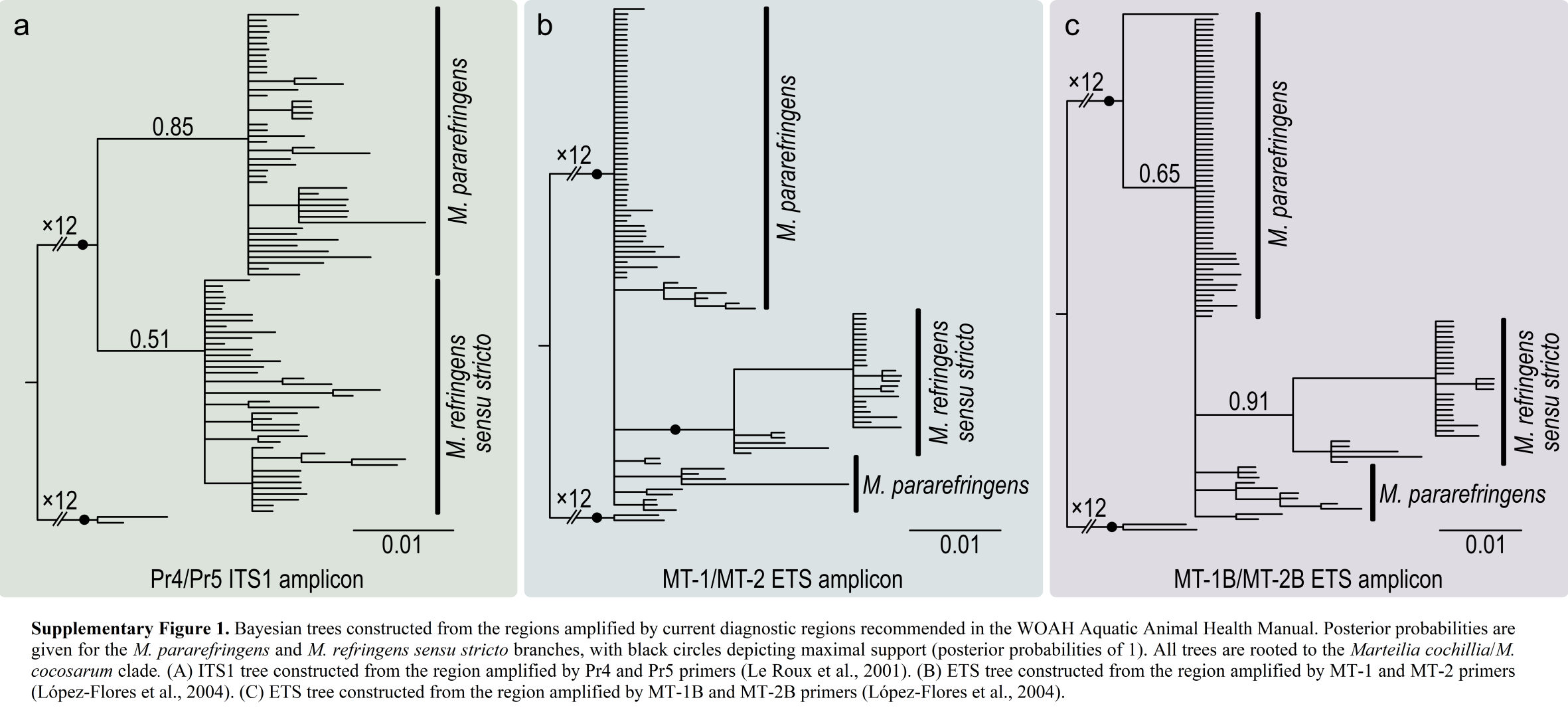

Supplement: Hooper et al. supplementary material 1 — Hooper et al. supplementary material [file S0031182025100796sup001.tiff]
