# Supplementary material for: Full ribosomal RNA gene arrays confirm Marteilia refringens sensu stricto and Marteilia pararefringens as separate species, and assess the validity of current diagnostic regions
Source: Parasitology. 2025 Sep 8;152(12):1247–62. doi: 10.1017/S0031182025100796 (PMC12921245; doi:10.1017/S0031182025100796)
Supplement: Hooper et al. supplementary material 2 — Hooper et al. supplementary material [file S0031182025100796sup002.pdf]

Supplementary Table 1: Table outlining samples used in this study, including location sampled, sampling year, host species, *Marteilia* species, the regions of the *Marteilia* rRNA sequenced, the accession numbers associated with these regions, and average coverage of the two contigs produced to generate the full transcribed rRNA array. (*) indicates cloned sequences and ST=sequence type.

Country	Location on map	Sampling year	Host Species	Sample ID	*Marteilia* species determined by sequence analysis	Accession numbers of generated sequences			Illumina sequencing statistics	
						Full rRNA array (Assembled from Illumina reads)	ITS1 (Generated by PCR and Sanger Sequencing)	ETS (Generated by PCR and Sanger Sequencing)	Average coverage of the first rRNA array contig (x)	Average coverage of the second rRNA array contig (x)
Norway	A	2017	Plankton	17/23/1B	*M. pararefringens*	PP549160	-	-	276.23	161.68
				17/23/2C	*M. pararefringens*	PP549161	-	-	278.24	131.99
		2018	Plankton	18/20/21	*M. pararefringens*	PP549166	-	-	299.18	72.23
				18/20/22	*M. pararefringens*	PP549163	-	-	235.59	105.98
				18/20/23	*M. pararefringens*	PP549164	-	-	109.27	74.47
		2018	*My. edulis*	18/30/06	*M. pararefringens*	PP549168	-	-	2858.81	3105.66
				18/30/07	*M. pararefringens*	PP549170	-	-	1885.58	1319.66
				18/30/15	*M. pararefringens*	PP549171	-	-	1869.34	1856.37
				18/30/17	*M. pararefringens*	PP549172	-	-	2504.59	1352.09
				18/30/20	*M. pararefringens*	-	ST1: PP549164	ST2: PP549181	-	-
				18/30/24	*M. pararefringens*	PP549169	-	-	751.41	1707.94
				18/30/27	*M. pararefringens*	PP549175	-	-	2104.13	1669.41
		2018	Plankton	18/31/54	*M. pararefringens*	-	ST1: PP549164	-	-	-
				18/31/55	*M. pararefringens*	-	ST1: PP549164	-	-	-
		2018	*My. edulis*	18/35/02	*M. pararefringens*	PP549176	-	-	1908.15	1576.53
				18/35/03	*M. pararefringens*	PP549177	-	-	3083.41	1630.54
				18/35/05	*M. pararefringens*	-	PP544142	ST2: PP549181	-	-
				18/35/08	*M. pararefringens*	-	ST22: PP549166	ST2: PP549181	-	-
				18/35/12	*M. pararefringens*	PP549162	-	-	351.51	1884.70
				18/35/14	*M. pararefringens*	-	ST10: PP544146	ST2: PP549181	-	-
				18/35/15	*M. pararefringens*	PP549173	-	-	2365.44	1821.76
				18/35/17	*M. pararefringens*	-	ST1: PP549164	ST2: PP549181	-	-
				18/35/21	*M. pararefringens*	PP549178	-	-	2359.43	841.42
				18/35/25	*M. pararefringens*	PP549174	-	-	1709.34	1468.67
				18/35/27	*M. pararefringens*	-	ST1: PP549164	-	-	-
				18/35/28	*M. pararefringens*	-	ST10: PP544146	ST2: PP549181	-	-
		2019	Plankton	19/24/107	*M. pararefringens*	PP549167	-	-	35.98	33.28
		2019	Plankton	19/33/111	*M. pararefringens*	PP549179	-	-	24.20	90.94
				19/33/112	*M. pararefringens*	PP549165	-	-	31.12	38.14
				19/33/113	*M. pararefringens*	PP549180	-	-	60.09	149.64
United Kingdom	B	2013	*My. edulis*	RA13082-26	*M. pararefringens*	-	ST2: PP544143	-	-	-
				RA13085-143	*M. pararefringens*	-	ST2: PP544143	PP549192	-	-
				RA13085-150	*M. pararefringens*	-	ST2: PP544143	PP549193	-	-
	C	2011	*My. edulis*	2011_119_07a	*M. pararefringens*	PP549158	-	-	198.08	125.87
				2011_119_15a	*M. pararefringens*	PP549159	-	-	183.39	222.63
	D	2006	*My. edulis*	2006_031_10	*M. pararefringens*	PP549157	-	-	181.10	261.07
				2006_031_11	*M. pararefringens*	PP549156	-	-	216.29	316.03
				2006_031_15	*M. pararefringens*	PP549153	-	-	277.92	285.55
		2008	*My. edulis*	2008_054_01	*M. pararefringens*	-	ST9: PP549158	-	-	-
				2008_054_09	*M. pararefringens*	PP549154	-	-	215.38	487.59
		2011	*O. edulis*	2011_117_25	*M. refringens*	PP549152	-	-	155.25	123.02
		2012	*O. edulis*	2012_145_01	*M. refringens*	PP549151	-	-	187.10	113.78
				2012_145_03	*M. refringens*	-	ST3: PP544144	PP549195	-	-
				2012_145_04	*M. refringens*	PP549150	-	-	225.07	243.21
		2013	*O. edulis*	2013_020_04	*M. refringens*	-	ST3: PP544144	-	-	-
				2013_020_35	*M. refringens*	-	ST3: PP544144	PP549197	-	-
		2013	*O. edulis*	2013_035_19	*M. refringens*	-	ST3: PP544144	-	-	-
				2013_035_31	*M. refringens*	PP549147	-	-	34.53	36.37
		2013	*O. edulis*	2013_036_24	*M. refringens*	-	ST3: PP544144	-	-	-
				2013_036_26	*M. refringens*	-	ST3: PP544144	-	-	-

		2013	*O. edulis*	2013_036_31	*M. refringens*	-	ST3: PP544144	-	-	-
		2015	*My. edulis*	2015_044_14	*M. pararefringens*	-	PP544153	-	-	-
		2017	*O. edulis*	2017_137_11	*M. refringens*	-	-	PP549196	-	-
France	E	2019	*O. edulis*	2019_036_01	*M. refringens*	PP549148	-	-	178.77	194.79
				2019_036_11	*M. refringens*	-	ST3: PP544144	PP549198		
				2019_036_07	*M. refringens*	PP549149	-	-	194.89	92.77
				2019_036_08	*M. refringens*	PP549145	-	-	252.61	231.84
	F	2007	*My. galloprovincialis*	2007_095_08	*M. pararefringens*	-	ST2: PP544143	-	-	-
	G	2003	*My. galloprovincialis*	2003_107_01	*M. pararefringens*	-	ST2: PP544143	-	-	-
		2007	*My. galloprovincialis*	2007_058_04	*M. pararefringens*	-	ST2: PP544143	PP549189	-	-
				2007_058_11	*M. pararefringens*	-	ST2: PP544143	-	-	-
		2007	*My. galloprovincialis*	2007_059_08	*M. pararefringens*	-	ST2: PP544143	-	-	-
				2007_059_17	*M. pararefringens*	-	-	PP549194	-	-
				2007_059_21	*M. pararefringens*	PP549155	-	-	248.92	165.03
				2007_059_30	*M. pararefringens*	-	-	PP549186	-	-
		2007	*My. galloprovincialis*	2007_060_02	*M. pararefringens*	-	-	PP549191	-	-
				2007_060_04	*M. pararefringens*	-	-	ST8: PP549182	-	-
				2007_060_09	*M. pararefringens*	-	-	ST8: PP549182	-	-
				2007_060_15	*M. pararefringens*	-	ST2: PP544143	-	-	-
				2007_060_18	*M. pararefringens*	-	ST5: PP544145	PP549185	-	-
				2007_060_20	*M. pararefringens*	-	-	PP549184	-	-
				2007_060_23	*M. pararefringens*	-	PP544148	PP549187	-	-
				2007_060_30_C2	*M. pararefringens*	-	ST2: PP544143	-	-	-
		2008	*My. galloprovincialis*	2008_010_22	*M. pararefringens*	-	ST5: PP544145	PP549188	-	-
Spain	H	2018	*My. galloprovincialis*	CFW05	*M. pararefringens*	-	ST21: PP544135	PP549183	-	-
			My. galloprovincialis	CFW26	*M. pararefringens*	-	PP544136-41*	PP549190	-	-
Greece	J	2010	*My. galloprovincialis*	2010_130_5c	*M. pararefringens*	-	PP544152	-	-	-
Italy	I	2007	*O. edulis*	2007_129_2_3	*M. refringens*	-	PP544149	-	-	-
				2007_129_2_4	*M. refringens*	-	PP544150	-	-	-
				2007_129_2_8	*M. refringens*	-	PP544151	-	-	-
		2008	*O. edulis*	2008_073_1a	*M. refringens*	PP549144	-	-	1555.62	2109.68
				2008_073_2b	*M. refringens*	PP549146	-	-	11288.04	4881.76
