# Supplementary material for: Full ribosomal RNA gene arrays confirm Marteilia refringens sensu stricto and Marteilia pararefringens as separate species, and assess the validity of current diagnostic regions
Source: Parasitology. 2025 Sep 8;152(12):1247–62. doi: 10.1017/S0031182025100796 (PMC12921245; doi:10.1017/S0031182025100796)
Supplement: Hooper et al. supplementary material 3 — Hooper et al. supplementary material [file S0031182025100796sup003.pdf]

Supplementary Table 2: Table outling the condensed sequence types used for phylogenetic analysis of the internal transcribed spacer 1 (ITS1). Accession numbers in red text were generated by other studies. Sequence IDs and accession numbers in black text were generated in this study, with ITS1 sequences originating from long-range amplicons and high-throughput sequencing denoted with (*), and ITS1 sequences generated with the MartDBITS primer set (Kerr et al., 2018) denoted with (**).

ITS1 Sequence Type	Accession number/Sample ID																														
1 (Accession PP549164 3034 -3956 bp)	18/20/23*	19/33/112*	18/30/20**	18/31/54**	18/31/55**	18/35/17**	18/35/27**	17/23/2C*																							
2 (Accession PP544143)	2003_107_01**	2007_058_04_C1**	2007_058_11**	2007_059_08**	2007_060_15**	2007_060_30_C2**	2007_095_08**	RA13082_26**	RA13085_143**	RA13085_150**	CFW25 clone**																				
3 (Accession PP544144)	2012_145_03**	2013_020_04**	2013_020_35**	2013_035_19**	2013_036_24**	2013_036_26**	2013_036_31**	2019_036_11**																							
4	DQ426569	DQ426624																													
5 (Accession PP544145)	2007_060_18**	2008_010_22**																													
6	MH304629/MH329400	MH342044/MH342046																													
7	DQ426560	DQ426570																													
8	MH304632/MH329403	MH304631/MH329402																													
9 (Accession PP549158 3033 -3964 bp)	2011_119_07a*	2008_054_01**																													
10 (Accession PP544146)	18/35/14**	18/35/28**																													
11	DQ426563	DQ426586	DQ426593	DQ426602	DQ426603	DQ426604	DQ426626	DQ426627	DQ426628	DQ426629	DQ426642	DQ426636	DQ426557	DQ426634	DQ426558	DQ426559	DQ426571	DQ426572	DQ426573	DQ426574	DQ426588	DQ426608	DQ426609	DQ426610	DQ426623	DQ426630	DQ426633	DQ426589	DQ426615	DQ426591	DQ426590
12	DQ426561	DQ426562	DQ426592	DQ426594	DQ426599	DQ426600	DQ426601	DQ426614	DQ426637	DQ426638	DQ426606	DQ426556	DQ426605	DQ426607	DQ426548	DQ426579	DQ426580	DQ426643													
13	DQ426550	DQ426553	AB513427	DQ426566	DQ426568	DQ426554	KR149572	KR149573	DQ426581	MH304863	DQ426575	JQ898013																			
14	DQ426611	DQ426612	DQ426613	DQ426616	DQ426619	DQ426620	DQ426617																								
15	AJ629346	AJ629347	AJ629350	AJ629351	AJ629349	AJ629348																									
16	DQ426576	DQ426577	DQ426578	AJ629335																											
17	DQ426631	DQ426632																													
18	DQ426545	DQ426549																													
19	KR149571	KR149574	KR149570																												
20	KC462431	KC462433																													
21	MH304864	MH304866	DQ426551	KC462432	PP544135**																										
22 (Accession PP549166 3034 -3956 bp)	18/20/21*	18/35/08**	18/35/21**	19/33/113**																											
23 (Accession PP549171 3034 -3956 bp)	18/35/15*	18/20/21*	19/24/107*																												
24 (Accession PP549152 3039 -3965 bp)	2011_117_25*	2013_035_31*	2019_036_1*	2019_036_8*																											
25 (Accession PP549172 3034 -3956 bp)	18/30/17*	18/30/27*	19/33/113*																												
